# Soil labile organic carbon fractions mediate microbial community assembly processes during long‐term vegetation succession in a semiarid region

**DOI:** 10.1002/imt2.142

**Published:** 2023-10-22

**Authors:** Jingwei Shi, Lin Yang, Yang Liao, Jiwei Li, Shuo Jiao, Zhouping Shangguan, Lei Deng

**Affiliations:** ^1^ State Key Laboratory for Soil Erosion and Dryland Farming on the Loes Plateau Institute of Soil and Water Conservation, Chinese Academy of Science and Ministry of Water Resources Yangling Shaanxi China; ^2^ University of Chinese Academy of Sciences Beijing China; ^3^ College of Soil and Water Conservation Science and Engineering (Institute of Soil and Water Conservation) Northwest A&F University Yangling Shaanxi China; ^4^ College of Life Sciences Northwest A&F University Yangling Shaanxi China

## Abstract

Conceptual diagram for the labile organic carbon (OC) fractions mediating microbial assembly processes during long‐term vegetation succession.

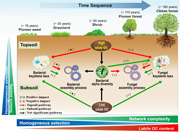

Owing to its sparse vegetation and low soil organic carbon (SOC) content, the Loess Plateau is one of the regions with the highest carbon (C) sequestration potential [1]. Vegetation succession is a widely adopted strategy for ecosystem recovery with the capacity to rehabilitate degraded lands and facilitate the sequestration of soil organic matter in a semiarid region [2, 3, 4, 5]. Additionally, it can influence the underground microbial community dynamics [6, 7]. The Ziwuling region has undergone approximately 160 years of secondary succession, making it a unique area with a complete sequence of natural vegetation succession following farmland abandonment on the Loess Plateau [4]. Thus, this region can provide us with a better understanding of the patterns of ecosystem changes during long‐term natural succession (Figure 1A and Supporting Information: Table S1). Considering the vital role of microorganisms in ecosystem function, examining the temporal changes of microbial communities during long‐term vegetation succession in a semiarid region is crucial [8].

**Figure 1:**
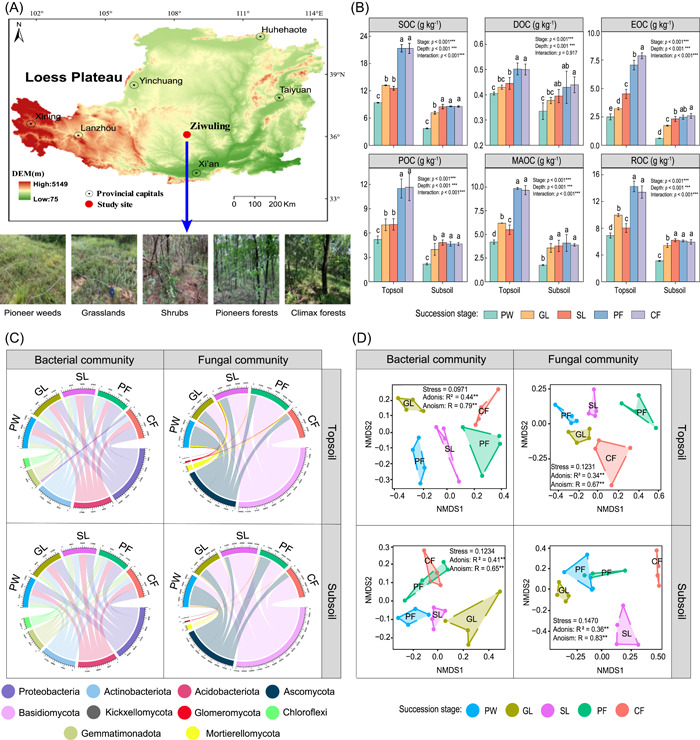
Sampling site locations and variations in soil organic C (SOC) fraction and microbial communities during long‐term vegetation succession. (A) The study sites on the Loess Plateau and photographs of the study site at each succession stage. (B) SOC and its fractions content in topsoil and subsoil as affected by long‐term vegetation succession. Different lowercase letters indicate significant differences at the successional stages (*p* < 0.05). Values present means ± standard error, *n* = 4. (C) Distribution of bacterial and fungal phyla in two soil depths during different succession stages. (D) Nonmetric multidimensional scaling (NMDS) ordination plot for bacterial and fungal communities during vegetation succession. The significances were examined using the Adonis and Anosim tests (**p < 0.01). CF, climax forests; DOC, dissolved organic carbon; EOC, easily oxidizable carbon; GL, grasslands; MAOC, mineral‐associated organic carbon; PF, pioneer forests; POC, particulate organic carbon; PW, pioneer weeds; ROC, recalcitrant organic carbon; SL, shrublands.

The assembly and composition of microbial communities are essential for ecosystem function [9, 10]. Deterministic and stochastic processes are the two primary ecological processes involved in microbial assembly [11]. Traditionally, microbial community assembly has been regarded as being primarily influenced by deterministic processes, aligning with the principle that, “everything is everywhere, but the environment selects” [12]. Nevertheless, stochastic processes should not be disregarded when considering random extinctions or dispersal events [13]. Land use type also has a notable effect on assembly processes [14, 15, 16]. Stochastic processes have a greater influence on agricultural fields, whereas their impact diminishes in forested soils, and dispersal is vital for bacterial community dynamics in temperate grasslands [16]. Furthermore, there are environmental differences between the topsoil (0–20 cm) and subsoil (20–40 cm) in terrestrial ecosystems [17]. Previous studies have indicated that stochastic processes dominate community assembly, with drift prevailing in the topsoil and dispersal limitation playing a dominant role in the subsoil in the subtropical paddy soils [18]. This indicates that there are differences and associations between microbial characteristics in different spatial distributions, which may affect ecosystem function. Although extensive research has been conducted on the microbial assembly process [12, 19, 20], uncertainties about the community composition and assembly process of microbial in the topsoil and subsoil remain owing to different resource characteristics during vegetation succession.

The balance between assembly processes is strongly influenced by abiotic factors including soil carbon (C) and nitrogen [19, 21, 22]. Research on salt marsh plant community succession has shown that temporal fluctuations in SOC exert the strongest selective pressure on bacterial assemblies [23]. Among the eight soil indicators, SOC had the greatest impact on microbial assembly [24]. SOC is commonly conceptualized as a labile and stable C fraction in C storage models [25], and easily oxidizable carbon (EOC), particulate organic carbon (POC), and dissolved organic carbon (OC) are considered labile OC fractions that serve as early indicators of SOC variation [26, 27]. In contrast, recalcitrant organic carbon (ROC) and mineral‐associated organic carbon OC have highly stable OC fractions [28]. Owing to the functional diversity of SOC, many of these studies have predominantly focused on the association between labile SOC fractions and microbial community composition [26, 29, 30], leaving the relationship between SOC fractions and the assembly process relatively unclear. Within the framework of deterministic and stochastic processes, the impact of biotic factors on microbial community assembly cannot be overlooked, which could determine the functional attributes or niche occupancy of microbial communities [12, 31]. Keystone taxa are highly connected taxonomic groups that play crucial roles in the assembly process and functionality of microbial communities [32]. For example, *Sulfuricella*, *Rhodobacter*, and *Comamonadaceae*, drive microbial assembly processes mediated by graphene derivatives [33]. However, the keystone taxa that drive the microbial community assembly remain largely unknown in natural ecosystems, and enhancing understanding of this is crucial for comprehending the relationship between microbial diversity and functionality.

To address these issues, the topsoil (0–20 cm) and subsoil (20–40 cm) were selected during the progression of vegetation succession to explore the microbial community structure, co‐occurrence networks, assembly processes, and their relationships with SOC fractions. We hypothesized that: (1) deterministic processes are stronger in the subsoil than in the topsoil because of nutrient limitations, and (2) given that labile OC represents a readily usable energy source for microorganisms, it is likely that community assembly is mainly governed by labile OC fractions.

## RESULTS

### The content of SOC fractions during long‐term vegetation succession

Long‐term vegetation succession significantly increased the accumulation of SOC fractions in 0–40 cm soils from the pioneer weed stage onwards (*p* < 0.001; Figure 1B). The C content in the topsoil was significantly higher than that in the subsoil in all fractions and succession stages (*p* < 0.001; Figure 1B). After the shrubland stage, the content of SOC fractions significantly increased compared to that in the early succession stage (Figure 1B). When succession reached the pioneer forest stage (>110 years), the C content gradually stabilized (Figure 1B). Succession age and soil depth significantly affected SOC and its fractions (*p* < 0.001; Figure 1B).

### Soil microbial communities and assembly processes

The bacterial communities primarily comprised the phyla Proteobacteria, Actinobacteria, Acidobacteria, Gemmatimonadota, and Chloroflexi (Figure 1C). The phyla Basidiomycota, Ascomycota, Mortierellomycota, Glomeromycota, and Kixellomycota were dominant in the fungal communities (Figure 1C). The abundances of Proteobacteria, Actinobacterta, Gemmatimonadota, and Mortierellomycota were significantly higher in the topsoil than in the subsoil, whereas those of Chloroflexi, Basidiomycota, and Ascomycota were lower (*p* < 0.05; Supporting Information: Figure S1). The relative abundances of Proteobacteria, Gemmatimonadota, and Chloroflexi were not significantly affected by the long‐term vegetation succession in the subsoil (*p* < 0.05; Supporting Information: Figure S1). Overall, long‐term vegetation succession decreased the alpha diversity of bacterial and fungal communities, with the Chao 1 and Shannon indices in the topsoil being higher than those in the subsoil (Supporting Information: Figure S2). A combination of NMDS analysis, ANOSIM, and Adonis statistical tests indicated that microbial communities varied strongly in composition with succession stage (Figure 1D). Soil depth also significantly affected the microbial communities (Supporting Information: Tables S2 and S3).

**Figure 2:**
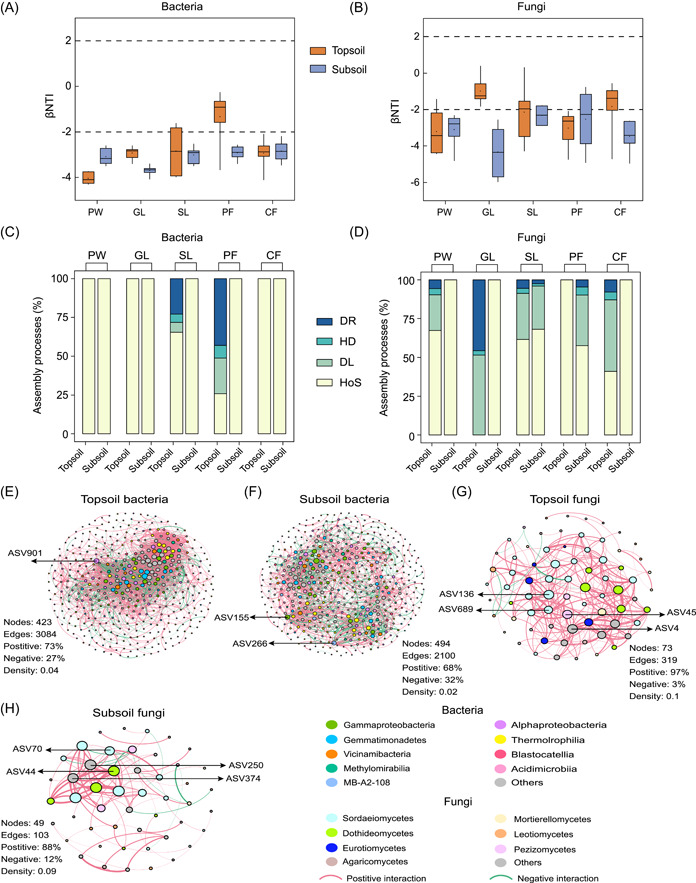
Assembly processes and co‐occurrence patterns of the microbial community. β nearest taxon index (βNTI) in bacteria (A) and fungi (B) across the succession stage. Contributions of ecological assembly processes dominating bacterial (C) and fungal (D) community turnover. The dotted lines mark the positions of 2 and −2. (E–H) The co‐occurrence networks of microbial communities in the topsoil and subsoil. The network is colored by class. The node (amplicon sequence variants [ASVs]) sizes are proportional to the connection number. Only nodes that were significantly (*p* < 0.05) and strongly (Spearman's > 0.7) correlated with each other were connected (edges). The thickness of the edge between two nodes is proportional to the value of Spearman's correlation coefficients. Red and green edges indicate positive and negative interactions between two individual nodes, respectively. The nodes are labeled with the keystone taxa associated with βNTI; Detailed information is provided in Supporting Information Table S5. CF, climax forests; DL, dispersal limitation; DR, drift; GL, grasslands; HD, homogenizing dispersal; HoS, homogeneous selection; PF, pioneer forests; PW, pioneer weeds; SL, shrublands.

The assembly processes along vegetation succession across the two soil depths were investigated using null model analyses. The results showed that deterministic processes (|βNTI| > 2), especially homogeneous selection, were critical for the community assembly of bacteria and fungi at both depths (Figure 2A–D). Furthermore, the deterministic assembly processes of the microbial community in the topsoil were weaker than those in the subsoil. Overall, the deterministic processes (homogeneous selection) of the microbial community in later succession stages were weaker compared to early stages (Figure 2A,C). Drift and dispersal limitations were critical for the bacterial and fungal stochastic processes at both depths (Figure 2B,D).

### Co‐occurrence networks analysis

The bacterial and fungal networks showed distinct patterns at each depth (Figure 2E–H). Edges and the average degree decreased with soil depth, demonstrating that the co‐occurrence network of the topsoil was more complex than that of the subsoil (Table S4). Additionally, there were more positive correlations in the topsoil than in the subsoil (Figure 2E–H). The cross‐kingdom network of the microbial community across the two soil depths with succession was further explored (Supporting Information: Figure S3), and it was found that the network nodes and edges among the taxa increased from pioneer weed to climax forests (Supporting Information: Table S4), suggesting that long‐term vegetation succession affected microbial associations and increased the complexity of the microbial networks.

**Figure 3:**
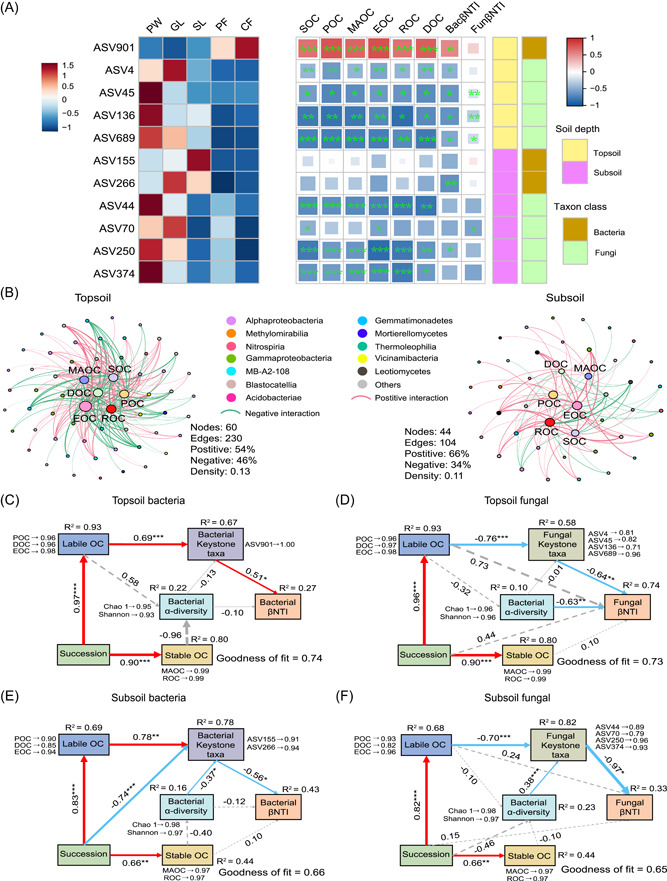
Drivers of microbial assembly processes during long‐term vegetation succession. (A) Relative abundance of keystone taxa and the correlation with soil organic carbon fractions and βNTI. The left heatmap shows the relative abundance (*Z* scores) of keystone taxa in various succession stages. The right heatmap shows the Spearman correlations between keystone taxa, soil organic C (SOC) fractions, and βNTI. Red and blue colors indicate positive and negative correlations, respectively. *p < 0.05, **p < 0.01, ***p < 0.001. (B) Taxon–soil organic carbon fractions networks of microbial communities in topsoil and subsoil. Nodes represented amplicon sequence variants (ASVs), and the sizes of nodes are proportional to the number of connections. Edges indicate a connection with a strong and significant (*p* < 0.05) correlation. (C–F) PLS‐PM showed the cascading relationships of succession times, SOC fractions, bacterial diversity, keystone taxa, and assembly of bacteria and fungi in two soil depths. Red and blue arrows indicate positive and negative effects (*p* < 0.05), respectively. The gray arrow indicates that the effect is not significant. The numbers on the arrows represent standardized path coefficients. The width of the arrows is proportional to the strength of the path coefficients. *R* ^2^ indicates the proportion of variance explained. Models were assessed by a goodness of fit statistic. BacβNTI, the β‐nearest taxon index of the bacterial community; CF, climax forests; DOC, dissolved organic carbon; EOC, easily oxidizable carbon; FunβNTI, the β‐nearest taxon index of the fungal community; GL, grasslands; MAOC, mineral‐associated organic carbon; OC, organic carbon; PF, pioneer forests; PLS‐PM, partial least squares path model; POC, particulate organic carbon; PW, pioneer weeds; ROC, recalcitrant organic carbon; SL, shrublands. *p < 0.05, **p < 0.01, ***p < 0.001.

Network analysis also identified the genus *Dongia* (ASV901) as a keystone amplicon sequence variant (ASV) in topsoil bacteria, and the genera *MND1* (ASV155) and *MB‐A2‐108* (ASV266) in the subsoil (Figure 2E,F and Supporting Information: Table S5). The keystone ASVs in topsoil fungi included the phyla *Ascomycota* (ASV4), the family *Pezizaceae* (ASV45), the genus *Fusarium* (ASV136), and the class *Sordarioclamycetes* (ASV689) (Figure 2G and Supporting Information: Table S5). The genera *Alternaria* (ASV44), *Fusarium* (ASV70), *Filobasidium* (ASV374), and the order *Chaetothyriales* (ASV250) were identified as keystone ASVs in subsoil fungi (Figure 2H and Supporting Information: Table S5). Overall, the relative abundance of keystone taxa decreased during long‐term succession and was negatively correlated with SOC fractions and assembly processes (Figure 3A). It is worth noting that the relative abundance of ASV901 increased with succession and showed a positive correlation with SOC fractions and assembly processes (Figure 3A).

### Linking SOC fractions and microbial community to assembly processes

The taxon–environment network indicates the relationship between SOC fractions and microbial community. The topsoil network consisted of 60 nodes and 230 edges (Figure 3B), while the subsoil network comprised 44 nodes and 104 edges (Figure 3B). EOC (connection number: 42) was the most important fraction that was closely associated with microbial taxa in topsoil (Figure 3B). EOC, POC, and ROC (with connection numbers 20, 20, and 21, respectively) were closely associated with the taxa in the subsoil (Figure 3B).

The linear regression model showed that SOC fractions were negatively correlated with microbial alpha diversity (Supporting Information: Figure S4). SOC fractions more strongly reflected variations in fungal alpha diversity compared to bacteria. Furthermore, SOC fractions more strongly reflected both variations in the microbial community and assembly process compared to SOC, with labile OC providing the most suitable explanation (Supporting Information: Figures S5 and S6). These results have demonstrated that labile OC fractions more effectively explained the variation in the microbial community than SOC and stable OC. Therefore, SOC fractions were used as mediators to illustrate the impacts of vegetation succession on microbial assembly using the partial least squares path model (PLS‐PM) (Figure 3C–F).

The labile OC fractions were the main drivers of the assembly process and regulated this effect by affecting microbial traits (Figure 3C–F). The total effect of keystone taxa (0.51) on bacterial assembly was the highest in the topsoil (Supporting Information: Figure S7A), and was the highest for bacterial assembly in the subsoil (−0.56) (Supporting Information: Figure S7C). Bacterial alpha diversity impacted fungal community assembly (Figure 3D,F and Supporting Information: Figure S12). The total effects of labile OC fractions (0.73) and fungal keystone taxa (−0.97) were highest for fungal assembly in the topsoil and subsoil, respectively (Supporting Information: Figure S7B,D).

## DISCUSSION

Microbial communities use various life strategies to generate their responses, affecting SOC dynamics [34]. Based on their mineralization capacity and growth rates, microorganisms can be classified into r‐ (copiotrophic, fast‐growing) and K‐strategists (oligotrophic, slow‐growing) [35]. In this study, long‐term vegetation succession shifted the microbial communities from r‐ to K‐strategists (Supporting Information: Figure S8), which is in agreement with previous studies [36, 37]. The increase in the ratio of fungi to bacteria and soil extracellular enzyme activity in the later stages of succession further corroborates this result (Supporting Information: Figures S9 and S10). This is because K‐strategists are predominantly fungal and primarily produce extracellular enzymes to degrade complex C [38, 39, 40]. An unstable nutrient environment was conducive to the rapid growth of r‐strategists in the early stages [40], and the accumulation of ROC in the later stages may be driven by the increase in K‐strategists [39]. This is because selected species reduce SOC mineralization and availability for SOC storage [41]. These results indicate that long‐term vegetation succession impacts the microbial community, shifts communities from r‐ to K‐strategists, and strengthens SOC stability.

The microbial network in the topsoil was more complex than that in the subsoil during the long‐term vegetation succession (Figure 2E–H). The topsoil had higher SOC and greater resources, resulting in a higher degree of ASV co‐occurrence [42, 43]. Ecological networks comprising higher ratios of negative correlations are more stable due to negative interactions decreasing oscillations in disturbed communities [44, 45]. Therefore, the bacterial network stability was higher than that of fungi. Furthermore, higher modularity revealed a stabilized network by limiting the influence of losing taxa to the community [46]. In this study, the subsoil network had more negative correlations and higher modularity than the topsoil network (Figure 2E,H and Supporting Information: Table S4), which suggests that network stability increased with soil depths. This is because the subsoil had fewer environmental perturbations to maintain community stability. The number of nodes, edges, ratios of negative correlations, and modularity across the two depths gradually increased with succession (Supporting Information: Table S4), indicating that the microbial network tended to stabilize and complex in the later stage.

The taxon–environment network showed that microbial communities were primarily associated with labile OC (Figure 3B). EOC was more strongly correlated with the microbial taxa at the two soil depths than with the other SOC fractions (Figure 3B). EOC is readily used by microbial mineralization and is generally used to indicate changes in early soil C pools [47]. Consequently, it was the main factor affecting the microbial communities. These results suggest that long‐term vegetation succession increases the complexity and stability of microbial networks and that microbial communities are primarily associated with labile OC.

Clarifying the microbial assembly processes is critical for understanding ecosystem diversity and function [48]. In this study, deterministic processes (homogeneous selection) dominated the microbial assembly at the two soil depths during long‐term vegetation succession (Figure 2A−D). The niche‐based theory states that deterministic processes dominate the community structure [14]. Stronger deterministic processes in a relatively extreme, low‐resource environment are a general phenomenon [13], which explains the greater deterministic (lower βNTI) in the early stages and subsoil and supports the first hypothesis. Homogeneous selection is usually determined by similar environmental conditions that exerted significant selective forces, suggesting that there was less variation in microbial community structure during long‐term succession than expected to happen by chance [49]. The deterministic processes decreased compared to the early stage, despite still dominating community assembly during long‐term succession, which was consistent with the current views, and stochastic processes increased with the advancement of resource supply under weak environmental selection [23]. Extreme pH acts as a strict environmental filter that results in phylogenetic clustering, regardless of successional age, and is considered a crucial abiotic factor in community assembly [50, 51]. Based on these views, we found that the difference in pH at various succession stages was small (Supporting Information: Figure S11), which is thought to have caused a slow shift from deterministic to stochastic processes. The importance of stochastic processes suddenly increased in succession (Figure 2A,B), which may be related to a significant increase in nutrients such as SOC [23].

The PLS‐PM model was used to further explore the driving mechanisms of microbial assembly during long‐term succession. The results demonstrated that labile OC was the main driving factor of community assembly, compared to stable OC (Figure 3C–F and Supporting Information: Figure S7), which confirmed the second hypothesis. This is because labile OC is an easily applied energy source for microorganisms and affects community structure and composition [26]. The results also illustrate that keystone taxa are essential controlling factors in the community assembly process (Figure 3C–F and Supporting Information: Figure S7), as they are strongly connected microorganisms that play essential roles in the microbial community [42]. Succession decreased the relative abundance of most keystone taxa (Figure 3A). This study has suggested that these keystone taxa may paramount importance in deterministic processes, but as stochastic processes increase, their importance gradually diminishes. The negative correlation between the keystone taxa and βNTI further supported this observation (Figure 3A). *Fusarium* and *Alternaria* are considered pathogenic fungi in the rhizosphere, and a decrease in their abundance benefits the maintenance of rhizosphere environmental stability [52, 53]. *Dongia* is recognized as a beneficial bacterium that can aid in pathogen resistance, such as *Xanthomonadaceae*, and ensure healthy plant growth [54]. The significant positive correlation between *Dongia* and bacterial βNTI suggests that it may play a crucial role in stochastic bacterial processes (Figure 3A). Overall, these keystone taxa play crucial roles in microorganisms and are the dominant factors driving community assembly.

Interactions between bacteria and fungi are common in the soil and play essential roles in facilitating ecological processes [12, 55]. This study found that soil bacteria impact the assembly of fungal communities (Figure 3C–F), and the correlation between fungal βNTI and bacterial community alpha diversity also supports this conclusion (Supporting Information: Figure S12). This may be attributed to the complex interactions between bacteria and fungi. Some volatiles released by soil bacteria have antagonistic effects on fungi [56], and bacteria facilitate fungal decomposition by providing nitrogen [57]. In summary, long‐term vegetation succession affects labile OC, regulating the alpha diversity of bacterial and keystone taxa and thereby controlling the assembly process.

This study has illustrated the relationship between SOC fractions, microbial communities, and assembly processes during long‐term vegetation succession. Given the key role of labile OC, the shift in assembly processes is determined by resource availability rather than succession sequences. This result is consistent with the neutral hypothesis [23], highlighting the non‐negligible role of labile OC in determining microbial community assembly during long‐term vegetation succession. This study has suggested that bacterial alpha diversity may drive fungal assembly. However, this was not directly tested in a laboratory, so further validation through controlled experiments is required to confirm this relationship. The keystone taxa identified in this study were involved in community assembly, providing a new targeted therapy and an indicator of the soil microenvironment. Keystone taxa should be further examined and cultured using metagenomic and metatranscriptomic approaches in future studies.

## CONCLUSION

This study investigated the relationship between microbial community assembly processes and SOC fractions during long‐term vegetation succession. The results show that homogeneous selection dominated bacterial and fungal community assembly processes during long‐term vegetation succession. The labile OC drove the microbial assembly processes by affecting bacterial alpha diversity and keystone taxa. In addition, soil bacteria were found to impact fungal community assembly. Taken together, this study provides new insights into the link between microbial communities and labile OC over long temporal scales.

## AUTHOR CONTRIBUTIONS

Jingwei Shi and Lin Yang conceived and supervised the study. Jingwei Shi and Jiwei Li established the experimental sites. Jingwei Shi and Yang Liao collected the samples and analyzed the data. Shuo Jiao, Zhouping Shangguan, and Lei Deng assisted with data analysis. Jingwei Shi wrote the manuscript with input from all authors.

## CONFLICT OF INTEREST STATEMENT

The authors declare no conflict of interest.

## Supporting information

Supporting information.

Supporting information.

## Data Availability

The data that support the findings of this study are available from the corresponding author upon reasonable request. The bacterial and fungal DNA sequences generated during this study are available from the National Center for Biotechnology Information's Gen‐Bank database under the project accession numbers PRJNA1022789 and PRJNA1023009. The data and scripts used are saved in GitHub https://github.com/Shijingweisjw/iMETA-R-code. Supporting Information: Materials (figures, tables, scripts, graphical abstract, slides, videos, Chinese translated version, and updated materials) may be found in the online DOI or iMeta Science http://www.imeta.science/
