# Supplementary material for: Soil labile organic carbon fractions mediate microbial community assembly processes during long‐term vegetation succession in a semiarid region
Source: Imeta. 2023 Oct 22;2(4):e142. doi: 10.1002/imt2.142 (PMC10989986; doi:10.1002/imt2.142)
Supplement: Supplementary file 1 — Supporting information. [file IMT2-2-e142-s001.docx]

**Supplementary Information**

**Soil labile organic carbon fractions mediate microbial community assembly processes during long-term vegetation succession in a semiarid region**

**Running title:** Labile organic carbon fractions mediate microbial community assembly processes

Jingwei Shi^1,2^, Lin Yang^1,2^, Yang Liao^1,2^, Jiwei Li^3^, Shuo Jiao^4^, Zhouping Shangguan^1,2,3^, Lei Deng^1,2,3*^

^1^State Key Laboratory for Soil Erosion and Dryland Farming on the Loes Plateau, Institute of Soil and Water Conservation, Chinese Academy of Science and Ministry of Water Resources, Yangling, Shaanxi 712100, China

^2^University of Chinese Academy of Sciences, Beijing 100049, China

^3^College of Soil and Water Conservation Science and Engineering (Institute of Soil and Water Conservation), Northwest A&F University, Yangling, Shaanxi 712100, China

^4^State Key Laboratory of Crop Stress Biology in Arid Areas, Shaanxi Key Laboratory of Agricultural and Environmental Microbiology, College of Life Sciences, Northwest A&F University, Yangling, Shaanxi 712100, China

^*^Corresponding author: Prof. Dr. Lei Deng

Address: No. 26 Xinong Road, Yangling, Shaanxi 712100, China

Phone: 086-29-87012884, Fax: 086-29-87012210

E-mail: leideng@ms.iswc.ac.cn

**Supplementary** **materials and methods**

**Study area and sampling**

This study was conducted on Lianjiabian Forest Farm (35°03′–36°37′N, 108°10′–109°18′E, 1211–1453 m above sea level), Gansu Province, China. This region covers an area of approximately 23,000 km^2^ and has a mean annual temperature of 10 °C [1]. The dominant soil type in the study area was cambisol. Arable land was abandoned in the 19th century when local inhabitants emigrated due to war. This area has undergone natural succession from farmland to climax forests over the past 160 years [2], and therefore natural vegetation at various succession stages can be observed in this area. The method for identifying the age of vegetation communities was described in a previous study [2].

Five succession stages were selected in this study: (1) pioneer weed stage (approximately 15 years), (2) grassland stage (approximately 30 years), (3) shrubland stage (approximately 60 years), (4) pioneer forest stage (approximately 110 years), and (5) climax forest stage (> 160 years) (Table S1). Four independent replicate plots were set up for each stage, at least 100 m apart and within a radius of 5 km. Each plot had a similar elevation, aspect, and slope. The plot sizes of the forest, shrub, and herbaceous communities were 20 × 20 m, 5 × 5 m, and 2 × 2 m, respectively. Bulk soil samples were collected from the two depths at the center and at the four corners of each plot after removing aboveground litter. Each sample was sieved through a 2-mm screen to remove roots and debris. Soil samples from the same depth were mixed with a composite sample from each plot. This resulted in 40 samples (2 soil depths × 5 succession stages × 4 independent plots) for subsequent analyses. The samples were then separated into three subsamples. One subsample was air-dried for physicochemical properties analyses, the second was refrigerated at 4 °C to measure dissolved organic C (DOC), while the third was stored at −80 °C before DNA extraction could take place.

**Assessment of soil organic carbon and fractions**

Soil organic C content was quantified as previously reported [2]. A total organic carbon analyzer (Shimadzu Corp, Kyoto, Japan) was used to determine the DOC content after extraction with 0.5 mol L^−1^ K_2_SO_4_. A wet sieve assembly was used to separate POC (≥ 53 μm) and MAOC (< 53 μm), after dispersing the soil particles in a sodium hexametaphosphate solution [3]. Easily oxidized carbon (EOC) was measured using the KMnO_4_ oxidation method, and the calculation of recalcitrant organic carbon (ROC) involved the subtraction of SOC and EOC [4].

**DNA extraction and bioinformatics analysis**

Total microbial DNA was extracted from soil samples using the cetyltrimethylammonium bromide method [5]. The V4–V5 and ITS1 regions of the 16S and 18S rRNA genes were targeted in order to profile the bacterial and fungal communities, respectively. The target sequences were amplified by a polymerase chain reaction using the primer pairs 515F/907R for bacteria and ITS5-1737F/ITS2-2043R for fungi [6,7]. Sequencing was conducted using the Nova Seq 6000 platform (Illumina Inc., San Diego, CA, USA). Reads were quality-filtered and assembled into amplicon sequence variants (ASVs) using the DADA2 module in QIIME2 (Version QIIME2-202006) [8]. For bacteria, the annotation databases were the Silva Database and the Unite Database for fungi [8]. The sequencing data were uploaded to the National Center for Biotechnology Information Sequence Read Archive database under accession number PRJNA1022789 and PRJNA1023009.

**Microbial community assembly analysis**

The β- nearest taxon index (βNTI) is commonly used to quantify community construction [9].

A null model was generated through 999 randomizations based on the observed data (ASV table and genetic development tree), and the βNTI was calculated as the deviation between the observed β-mean-nearest taxon distance (βMNTD) and the mean of the null βMNTD distribution in units of standard deviation [9]. If βNTI was > 2 then the βMNTD observed was either significantly smaller or larger than the βMNTD obtained from the stochastic simulation. This finding indicated that the change in the microbial community is predominantly as a result of deterministic processes, through which βNTI < -2 and βNTI > 2 represent homogeneous and heterogeneous selection, respectively. If |βNTI| was < 2, then the change in the microbial community was predominantly a result of stochastic processes. The βNTI combined with the Bray–Curtis‐based Raup–Crick (RCbray) were further used to determine the relative contribution of the assembly process with |βNTI| < 2. The relative contribution of dispersal limitation was estimated in terms of percentages of paired comparisons with |βNTI| < 2 and RCbray > 0.95. The contribution of relative homogenizing dispersal was estimated in terms of percentages of paired comparisons with |βNTI| < 2 and RCbray < -0.95. The contribution of relative drift was estimated in terms of percentages of paired comparisons with |βNTI| < 2 and |RCbray| ≤ -0.95 [9,10].

**Co-occurrence network analysis**

Networks were built using the “WGCNA” package in R software (V4.1.2) [11]. The *p*-values were adjusted using Benjamini and Hochberg false discovery rates [12,13]. Only ASVs with relative abundance > 0.001 were used in the analyses. Statistical correlations were identified when Spearman’s r > 0.7 and *p* < 0.05 and were then incorporated into the co-occurrence network construction [13]. The R package “igraph” was used to calculated network topological characteristics [13]. The node and edge numbers and average degree were used to evaluate network complexity in this study, with higher node and edge numbers and average degrees indicating greater network complexity [13,14]. The R package ‘mixOmics’ was used to compute the value importance in projection (VIP) of partial least squares (PLS) regression [15]. This served as a predictor to assess the importance of ASVs occurred in the network for βNTI [15]. ASVs with high degrees (top five in the network) and VIP values (VIP > 1) were selected as the keystone taxa [15−17]. Network visualization was performed using Gephi software. The cross-kingdom co-occurrence networks of bacteria and fungi across soil depths at each succession stage and the taxon-SOC fraction network were constructed using the same procedure.

**Statistical analysis**

Two-way ANOVA was used to analyze the between-subject effects of succession age and soil depth on the content of SOC fractions using SPSS software (V23.0, IBM, Armonk, NY, USA). The taxonomic distribution of microbial taxa at various succession stages at the phylum level was visualized using a Circos plot in R software (V4.1.2). The R package “vegan” was used to calculate the alpha diversity of the microbial communities [18]. The Bray–Curtis measure of microbial community dissimilarity was analyzed using QIIME 2 software (Version QIIME2-202006), and the results were visualized using a nonmetric multidimensional scaling (NMDS) analysis using the “metaMDS” function of the R package “vegan” [19]. To obtain robust results for NMDS, significance tests were performed using analysis of similarities (ANOSIM) and nonparametric multivariate analysis of variance (Adonis). The NMDS scores of the first axis were used to represent bacterial and fungal community structures. Spearman's correlation analysis was employed to evaluate the correlations among keystone taxa, SOC fractions, and microbial community assembly using the R package “corrplot”. Linear regressions were used to analyze the relationship between SOC fractions with microbial community and assembly processes using the R package “ggplot2”. The partial least squares path model (PLS-PM) was used to further explore the pathways whereby SOC fractions control microbial community assembly during vegetation succession, using the R package “plspm”.

**References**

1. Wang, Kaibo, Yongwang Zhang, Zhuangsheng Tang, Zhouping Shangguan, Fan Chang, Feng'an Jia, Yiping Chen, Xinhua He, Weiyu Shi, Lei Deng. 2019. “Effects of grassland afforestation on structure and function of soil bacterial and fungal communities.” *Science of the Total Environment* 676: 396–406. https://doi.org/10.1016/j.scitotenv.2019.04.259

2. Shi, Jingwei, Lei Deng, Anna Gunina, Sulaiman Alharbi, Kaibo Wang, Jiwei Li, Yulin Liu, Zhouping Shangguan, Yakov Kuzyakov. 2023. “Carbon stabilization pathways in soil aggregates during long-term forest succession: Implications from δ13C signatures.” *Soil Biology and Biochemistry* 180: 108988. https://doi.org/10.1016/j.soilbio.2023.108988

3. Six, J., K. Paustian, E. T. Elliott, C. Combrink. 2000. “Soil structure and organic matter I. Distribution of aggregate-size classes and aggregate-associated carbon.” *Soil Science Society of America Journal* 64: 681–689. https://doi.org/10.2136/sssaj2000.642681x

4. Vieira, F. C. B., C. Bayer, J. A. Zanatta, J. Dieckow, J. Mielniczuk, Z. L. He. 2007. “Carbon management index based on physical fractionation of soil organic matter in an acrisol under long-term no-till cropping systems.” *Soil and Tillage Research* 96: 195–204. https://doi.org/10.1016/j.still.2007.06.007

5. Yuan, Qing-Bin, Ya-Meng Huang, Wen-Bin Wu, Pengxiao Zuo, Nan Hu, Yong-Zhang Zhou, Pedro J. J. Alvarez. 2019. “Redistribution of intracellular and extracellular free & adsorbed antibiotic resistance genes through a wastewater treatment plant by an enhanced extracellular DNA extraction method with magnetic beads.” *Environment International* 131: 104986. https://doi.org/10.1016/j.envint.2019.104986

6. Degnan, Patrick H., Howard Ochman. 2012. “Illumina-based analysis of microbial community diversity.” *The ISME Journal* 6: 183–194. https://doi.org/10.1038/ismej.2011.74

7. Walters, William, R. Hyde Embriette, Donna Berg-Lyons, Gail Ackermann, Greg Humphrey, Alma Parada, A. Gilbert Jack, et al. 2015. “Improved bacterial 16S rRNA gene (V4 and V4-5) and fungal internal transcribed spacer marker gene primers for microbial community surveys.” *mSystems* 1: e00009. https://doi.org/10.1128/msystems.00009-15

8. Callahan, Benjamin J., Paul J. McMurdie, Michael J. Rosen, Andrew W. Han, Amy Jo A. Johnson, Susan P. Holmes. 2016. “DADA2: High-resolution sample inference from Illumina amplicon data.” *Nature Methods* 13: 581–583. https://doi.org/10.1038/nmeth.3869

9. Stegen, James C., Xueju Lin, Jim K. Fredrickson, Xingyuan Chen, David W. Kennedy, Christopher J. Murray, Mark L. Rockhold, Allan Konopka. 2013. “Quantifying community assembly processes and identifying features that impose them.” *The ISME Journal* 7: 2069–2079. https://doi.org/10.1038/ismej.2013.93

10. Ning, Daliang, Mengting Yuan, Linwei Wu, Ya Zhang, Xue Guo, Xishu Zhou, Yunfeng Yang, Adam P. Arkin, Mary K. Firestone, Jizhong Zhou. 2020. “A quantitative framework reveals ecological drivers of grassland microbial community assembly in response to warming.” *Nature Communications* 11: 4717. https://doi.org/10.1038/s41467-020-18560-z

11. Langfelder, Peter, Steve Horvath. 2008. “WGCNA: an R package for weighted correlation network analysis.” *BMC Bioinformatics* 9: 559. https://doi.org/10.1186/1471-2105-9-559

12. Benjamini, Yoav, Abba M. Krieger, Daniel Yekutieli. 2006. “Adaptive linear step-up procedures that control the false discovery rate.” *Biometrika* 93: 491–507. https://doi.org/10.1093/biomet/93.3.491

13. Qiu, Liping, Qian Zhang, Hansong Zhu, Peter B. Reich, Samiran Banerjee, Marcel G. A. van der Heijden, Michael J. Sadowsky, et al. 2021. “Erosion reduces soil microbial diversity, network complexity and multifunctionality.” *The ISME Journal* 15: 2474–2489. https://doi.org/10.1038/s41396-021-00913-1

14. Guseva, Ksenia, Sean Darcy, Eva Simon, Lauren V. Alteio, Alicia Montesinos-Navarro, Christina Kaiser. 2022. “From diversity to complexity: Microbial networks in soils.” *Soil Biology and Biochemistry* 169: 108604. https://doi.org/10.1016/j.soilbio.2022.108604

15. Wang, Xiaoyue, Chao Liang, Jingdong Mao, Yuji Jiang, Qing Bian, Yuting Liang, Yan Chen, Bo Sun. 2023. “Microbial keystone taxa drive succession of plant residue chemistry.” *The ISME Journal* 17: 748–757. https://doi.org/10.1038/s41396-023-01384-2

16. Banerjee, Samiran, Florian Walder, Lucie Büchi, Marcel Meyer, Alain Y. Held, Andreas Gattinger, Thomas Keller, Raphael Charles, Marcel G. A. van der Heijden. 2019. “Agricultural intensification reduces microbial network complexity and the abundance of keystone taxa in roots.” *The ISME Journal* 13: 1722–1736. https://doi.org/10.1038/s41396-019-0383-2

17. Yang, Lin, Lanlan Du, Weijia Li, Rui Wang, Shengli Guo. 2023. “Divergent responses of phoD- and pqqC-harbouring bacterial communities across soil aggregates to long fertilization practices.” *Soil and Tillage Research* 228: 105634. https://doi.org/10.1016/j.still.2023.105634

18. Jiao, Shuo, Weimin Chen, Gehong Wei. 2022. “Core microbiota drive functional stability of soil microbiome in reforestation ecosystems.” *Global Change Biology* 28: 1038–1047. https://doi.org/10.1111/gcb.16024

19. Jiao, Shuo, Haiyan Chu, Baogang Zhang, Xiaorong Wei, Weimin Chen, Gehong Wei. 2022. “Linking soil fungi to bacterial community assembly in arid ecosystems.” *iMeta* 1: e2. https://doi.org/10.1002/imt2.2

**Supplementary figure legends**

**Figure S1.** Relative abundance of the dominant bacterial (a-e) and fungal (f-h) phyla in topsoil and subsoil during vegetation succession. PW: pioneer weeds, GL: grasslands, SL: shrublands, PF: pioneer forests, CF: climax forests. Different lowercase letters indicate significant differences at the successional stages (*p* < 0.05).

**Figure S2.** Alpha diversity (a, c: Chao index; b, d: Shannon index) of microbial as affected by vegetation succession in two soil depth. AVE: the mean values of the two soil depths. One-way ANOVA was used to test for differences between successions, and all soil depths were tested by one sample t-test separately. Letters indicate significant differences (*p* < 0.05) among vegetation succession. * *p* < 0.05, ** *p* < 0.01 and *** *p* < 0.001 between the soil depths.

**Figure S3.** Cross-kingdom co-occurrence network of microbial communities cross the two soil depths during forest succession. The network is colored by phylum. The nodes (ASVs) size are proportional to the connection number. Only nodes that were significantly (*p* < 0.05) and strongly (Spearman’s > 0.7) correlated each other were connected (edges). The thickness of each edges between two nodes is proportional to the value of Spearman’s correlation coefficients. Red and green edges indicate positive and negative interactions between two individual nodes, respectively.

**Figure S4.** The relationships of microbial communities to SOC fractions in two soil depth. POC: particulate organic C, DOC: dissolved organic C, EOC: easily oxidized C, MAOC: mineral associated organic C, ROC: recalcitrant organic C.

**Figure S5.** The relationships of microbial community assembly processes to SOC fractions in two soil depth. BacβNTI: the β-nearest taxon index of bacterial community, FunβNTI: the β-nearest taxon index of fungal community.

**Figure S6.** The relationships of microbial alpha diversity to SOC fractions in two soil depth. POC: particulate organic C, DOC: dissolved organic C, EOC: easily oxidized C, MAOC: mineral associated organic C, ROC: recalcitrant organic C.

**Figure S7.** Standardized total effects of influence factors on bacterial community assembly in topsoil (a) and subsoil (c), and fungi assembly in topsoil (b) and subsoil (d) via partial least squares path model.

**Figure S8.** The ratio of k-strategist (Basidiomycota, Gemmatimonadota, Chloroflexi, and Acidobacterta) to r-strategist (Proteobacteria, Actinobacterta, Ascomycota, and Mortierellomycota).

**Figure S9.** The ratio of fungai to bacteria.

**Figure S10.** Soil extracellular enzymatic activity in topsoil and subsoil during vegetation succession. PW: pioneer weeds, GL: grasslands, SL: shrublands, PF: pioneer forests, CF: climax forests. Different lowercase letters indicate significant differences at the successional stages (*p* < 0.05).

**Figure S11.** Variation in soil pH among different successional stages. Values present means ± standard error, n = 4. Different lowercase letters indicate significant differences at the successional stages (*p* < 0.05).

**Figure S12.** Regression analysis of bacterial diversity and fungal assembly processes.


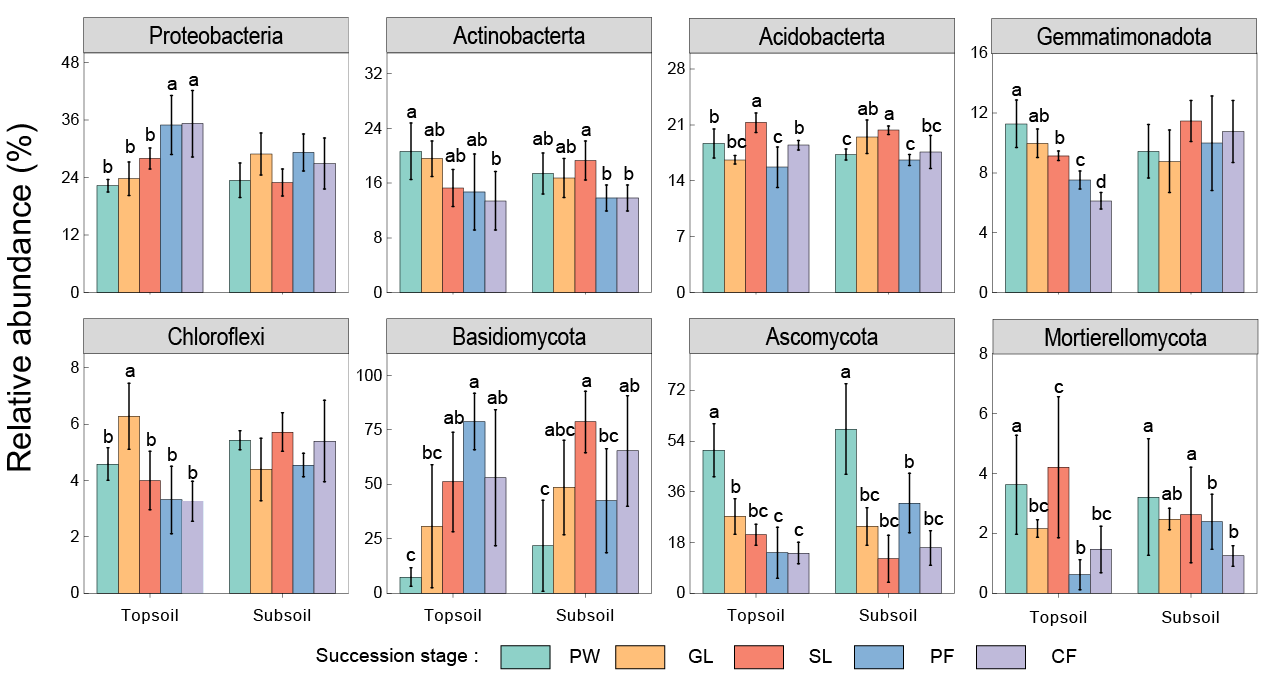


**Figure S1.** Relative abundance of the dominant phyla in topsoil and subsoil during vegetation succession. PW, pioneer weeds; GL, grasslands; SL, shrublands; PF, pioneer forests; CF, climax forests. Different lowercase letters indicate significant differences at the successional stages (*p* < 0.05).


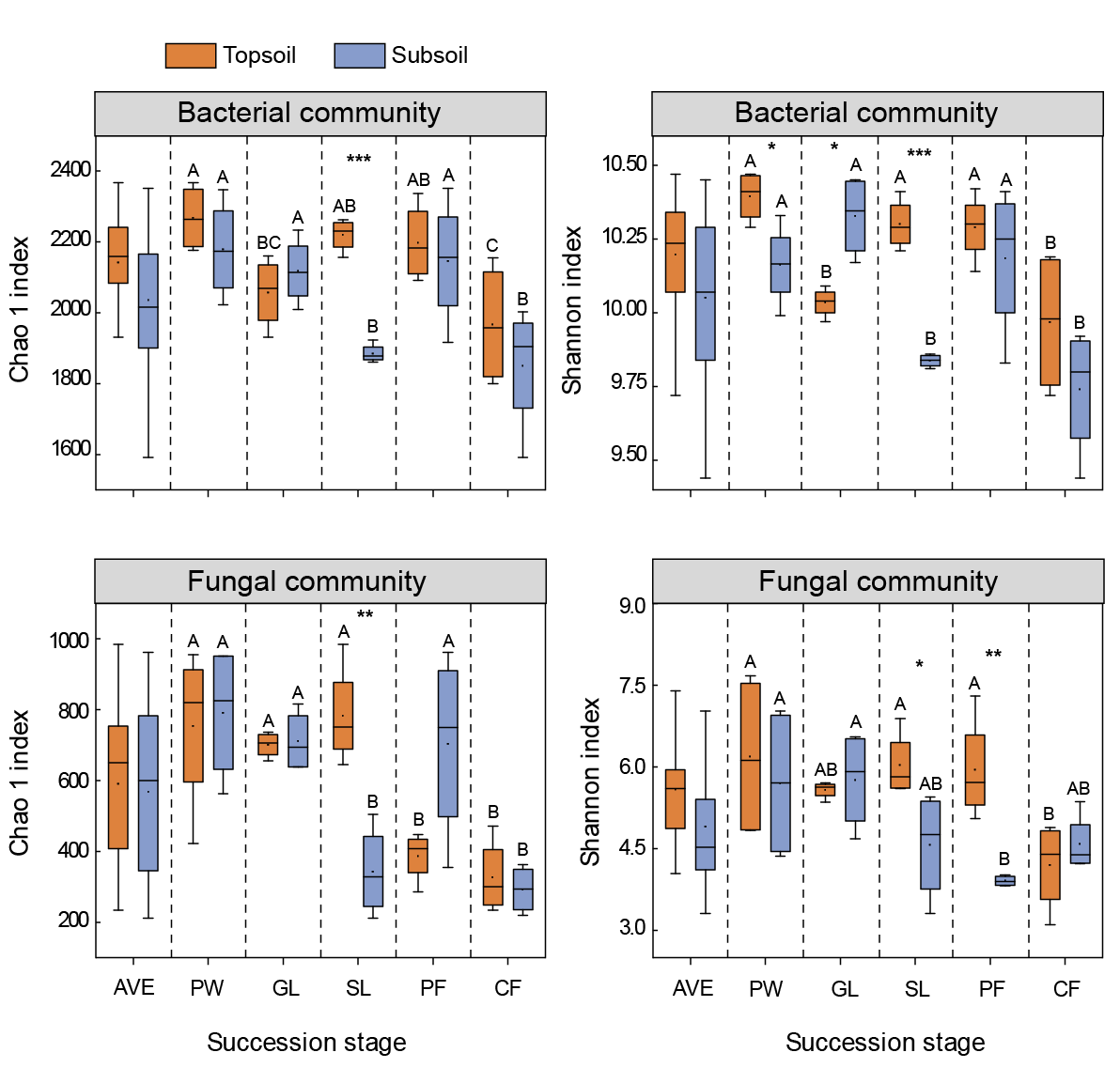


**Figure S2.** Alpha diversity of microbial as affected by vegetation succession in two soil depth. AVE, the mean values of the two soil depths. One-way ANOVA was used to test for differences between successions, and all soil depths were tested by one sample *t*-test separately. Letters indicate significant differences (*p* < 0.05) among vegetation succession. * *p* < 0.05, ** *p* < 0.01 and *** *p* < 0.001 between the soil depths.

**
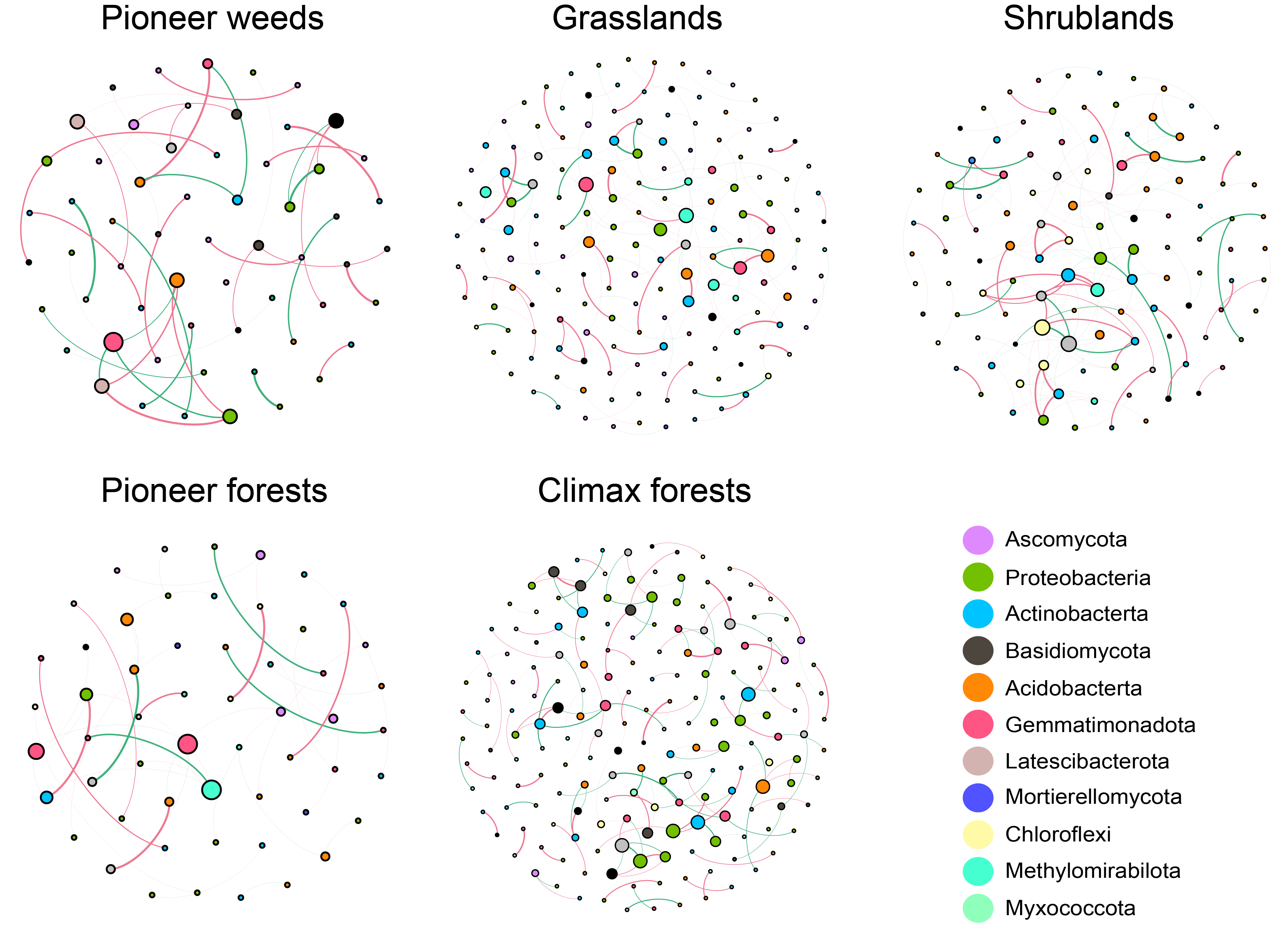
**

**Figure S3.** Cross-kingdom co-occurrence network of microbial communities cross the two soil depths during forest succession. The network is colored by phylum. The nodes (ASVs) size is proportional to the connection number. Only nodes that were significantly (*p* < 0.05) and strongly (Spearman’s > 0.7) correlated each other were connected (edges). The thickness of each edge between two nodes is proportional to the value of Spearman’s correlation coefficients. Red and green edges indicate positive and negative interactions between two individual nodes, respectively.


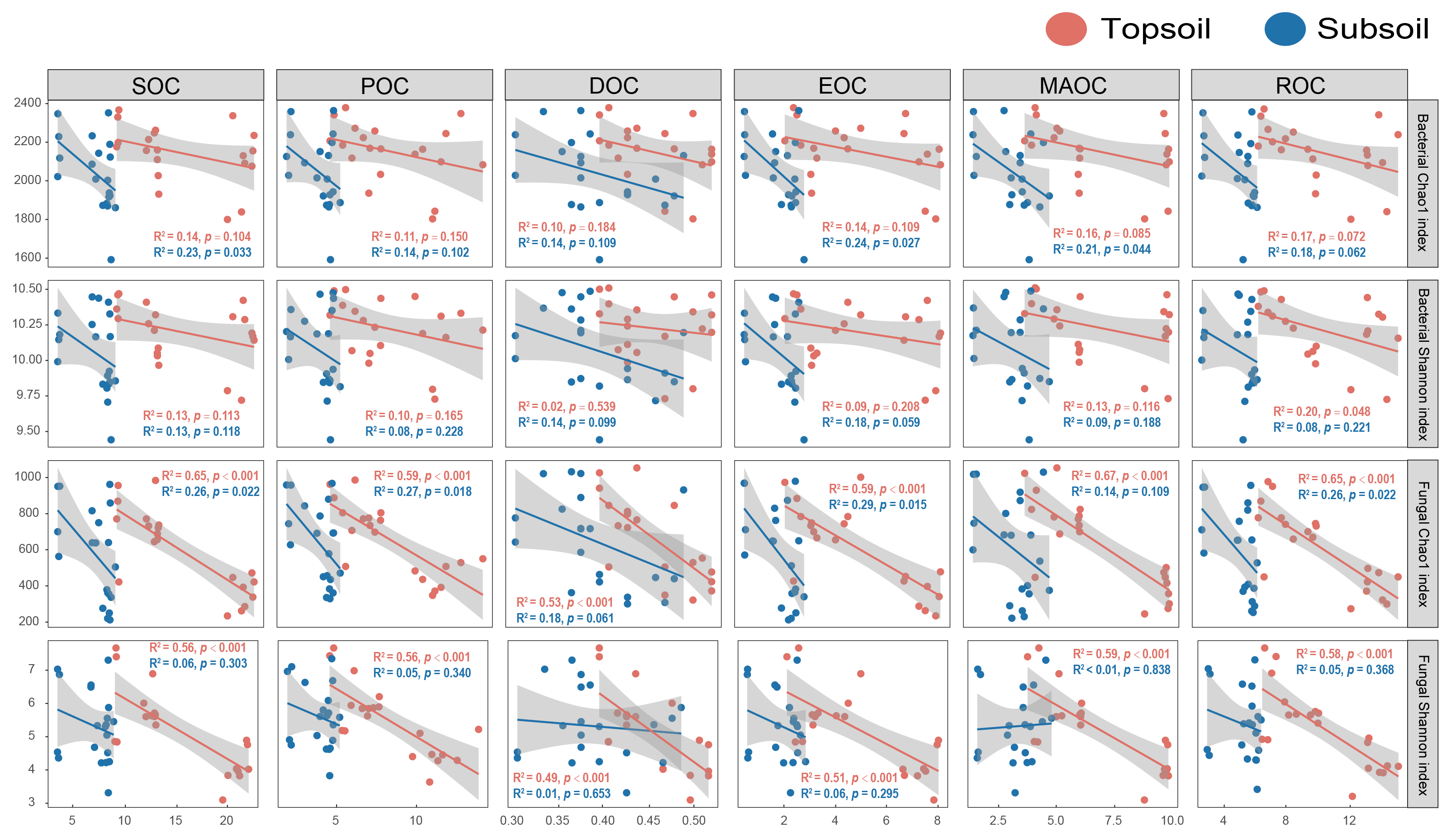


**Figure S4.** The relationships of microbial alpha diversity to SOC fractions in two soil depth. POC, particulate organic C; DOC, dissolved organic C; EOC, easily oxidized C; MAOC, mineral associated organic C; ROC: recalcitrant organic C.


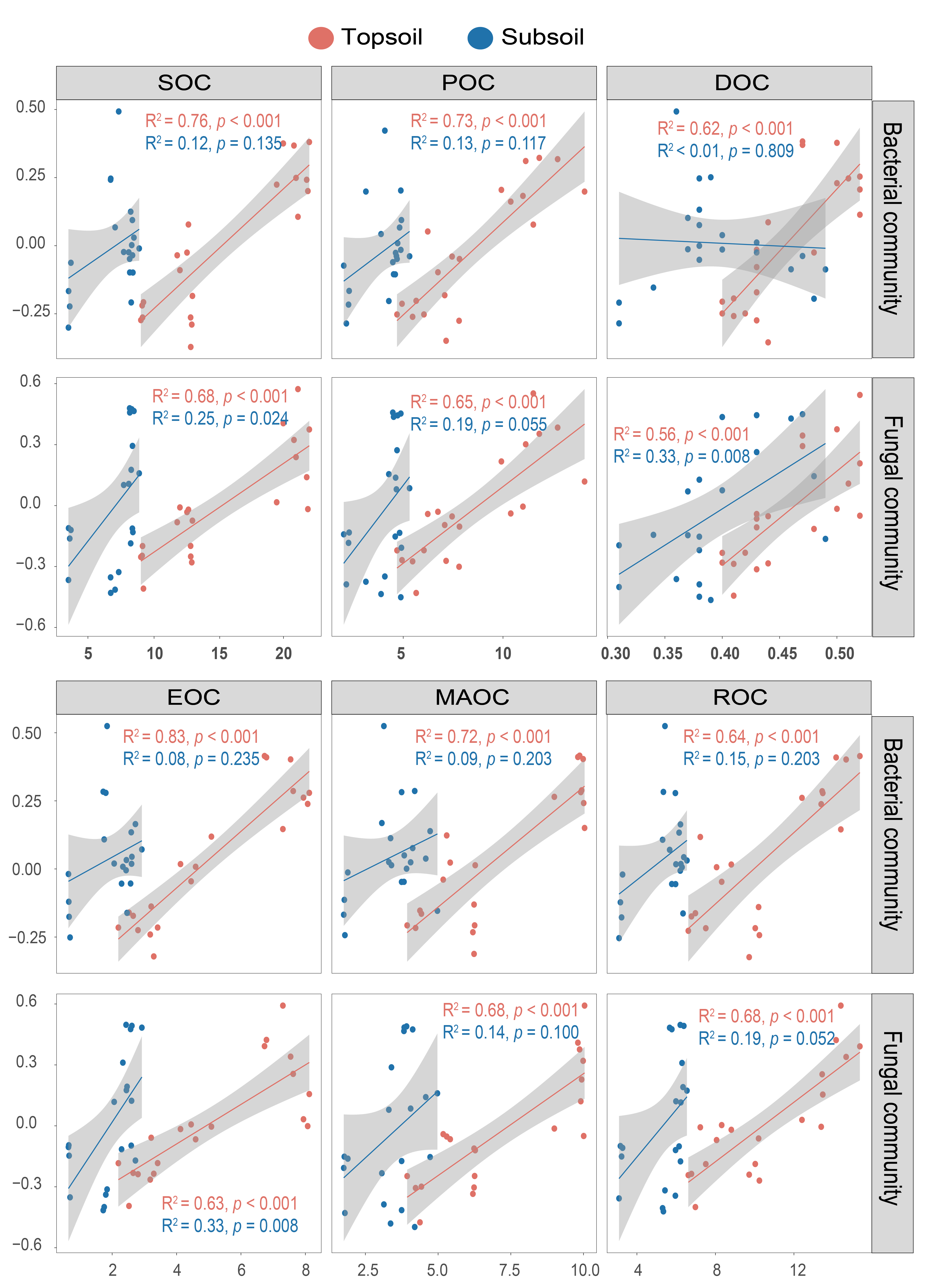


**Figure S5.** The relationships of microbial communities to SOC fractions in two soil depth. POC, particulate organic C; DOC, dissolved organic C; EOC, easily oxidized C; MAOC, mineral associated organic C; ROC, recalcitrant organic C.


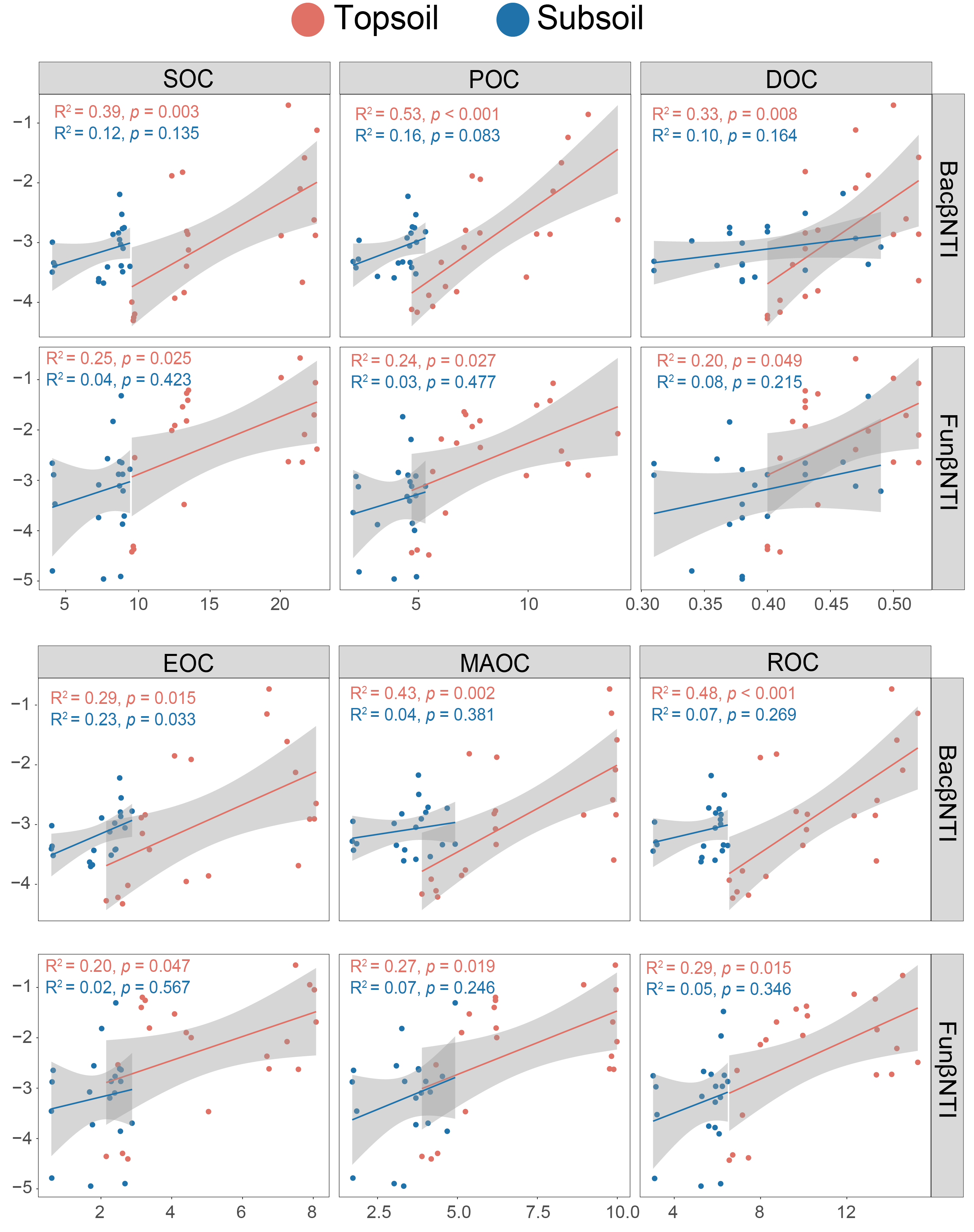


**Figure S6.** The relationships of microbial community assembly processes to SOC fractions in two soil depth. BacβNTI, the β-nearest taxon index of bacterial community; FunβNTI, the β-nearest taxon index of fungal community.


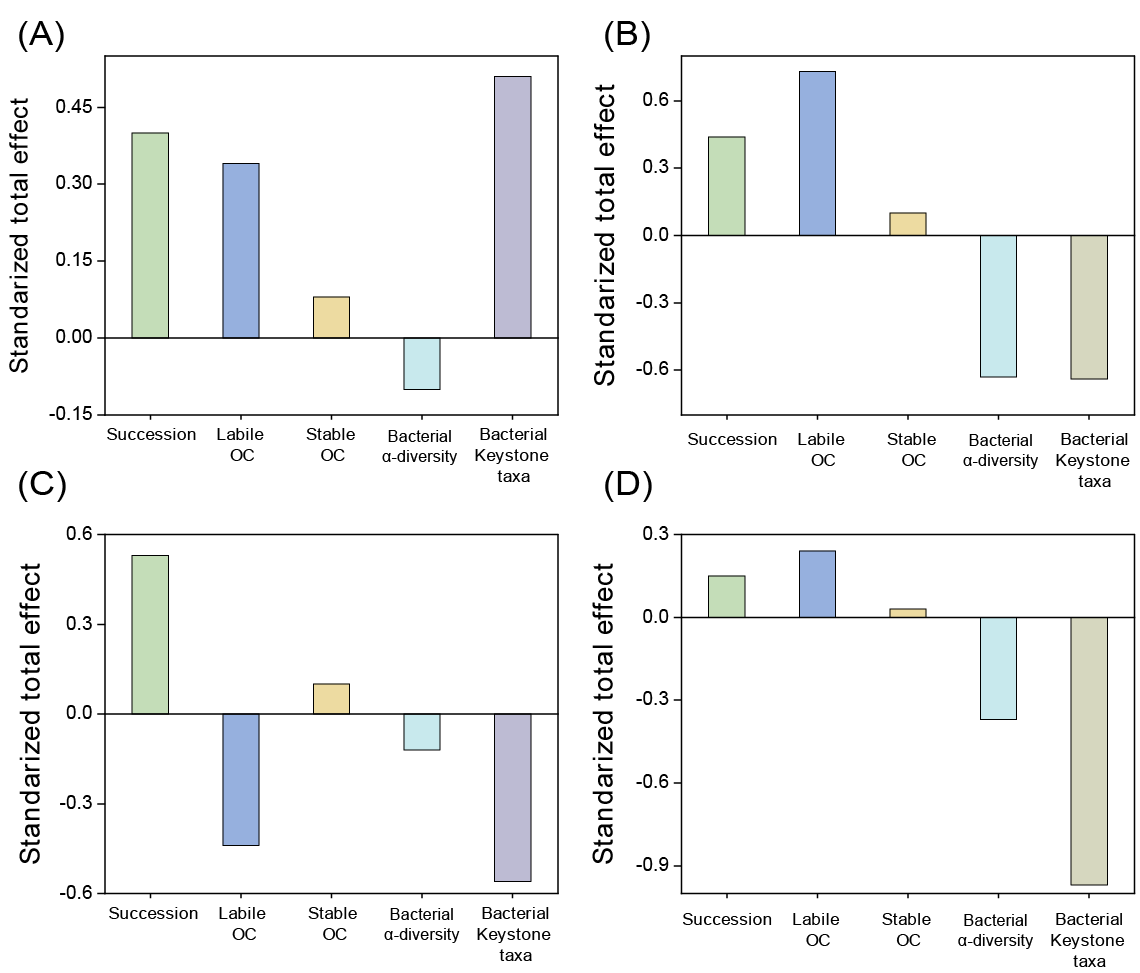


**Figure S7.** Standardized total effects of influence factors on bacterial community assembly in topsoil (A) and subsoil (C), and fungi assembly in topsoil (B) and subsoil (D) via partial least squares path model.

**
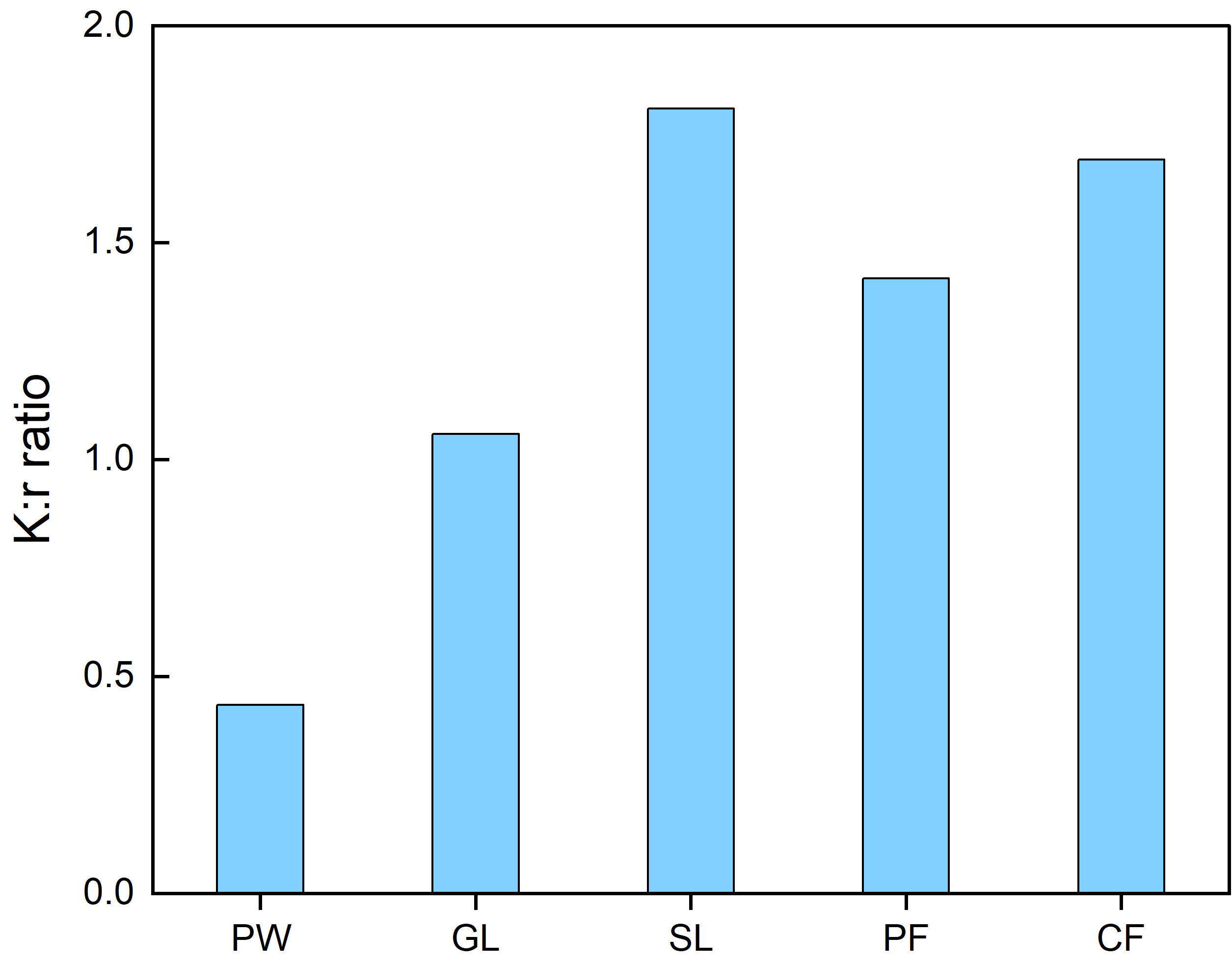
**

**Figure S8.** The ratio of k-strategist (Basidiomycota, Gemmatimonadota, Chloroflexi, and Acidobacterta) to r-strategist (Proteobacteria, Actinobacterta, Ascomycota, and Mortierellomycota).

**
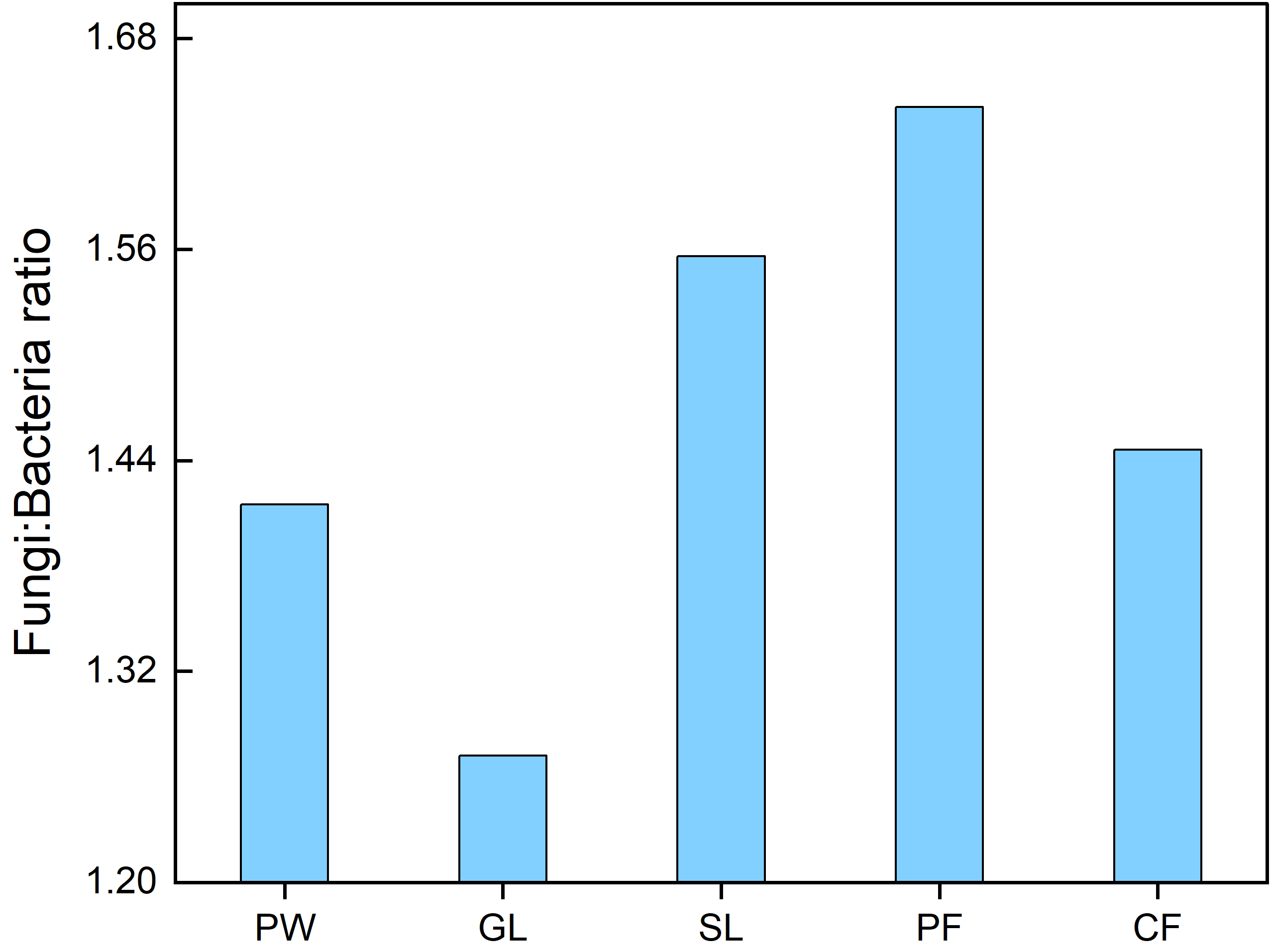
**

**Figure S9.** The ratio of fungi to bacteria.

**
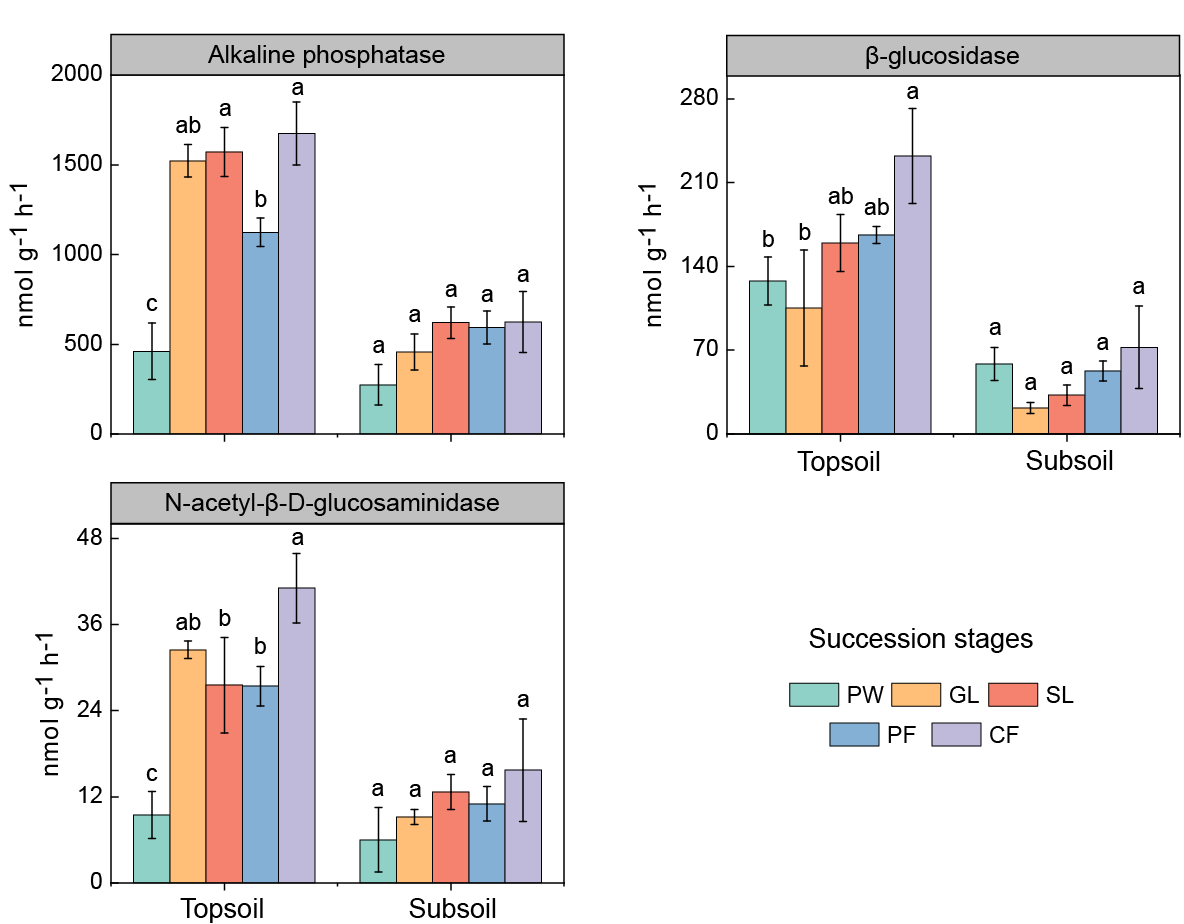
**

**Figure S10.** Soil extracellular enzymatic activity in topsoil and subsoil during vegetation succession. PW, pioneer weeds; GL, grasslands; SL, shrublands; PF, pioneer forests; CF, climax forests. Different lowercase letters indicate significant differences at the successional stages (*p* < 0.05).

**
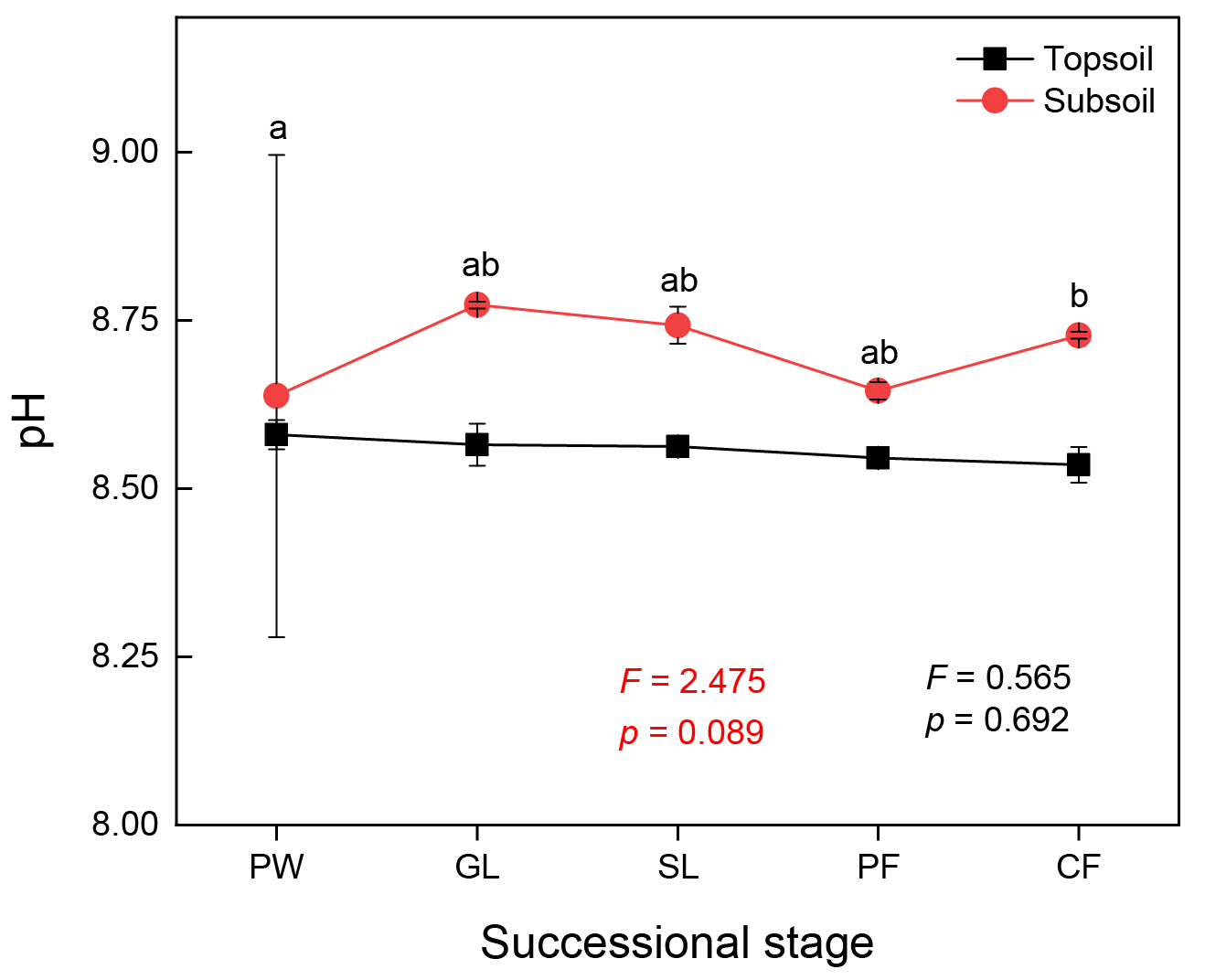
**

**Figure S11.** Variation in soil pH among different successional stages. Values present means ± standard error, n = 4. Different lowercase letters indicate significant differences at the successional stages (*p* < 0.05).

**
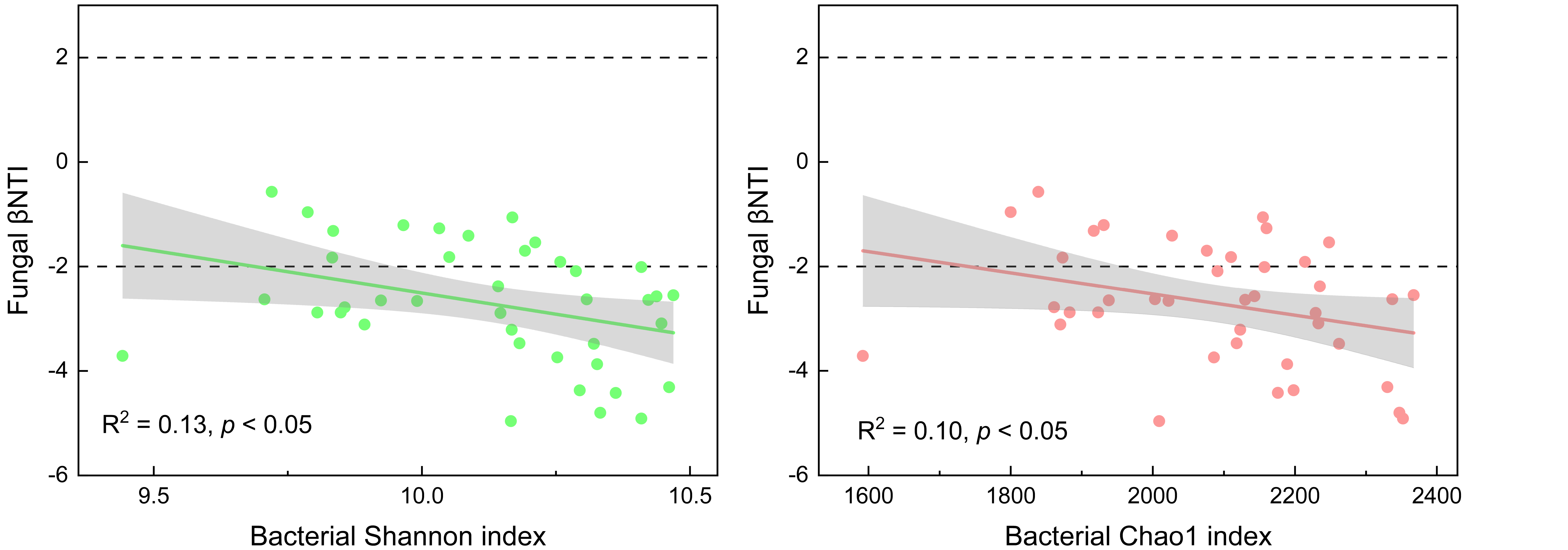
**

**Figure S12.** Regression analysis of bacterial diversity and fungal assembly processes.
